# Iodide of Potassium

**Published:** 1883-08-20

**Authors:** George Thomas Jackson


### IODIDE OF POTASSIUM.

BY GEORGE THOMAS JACKSON, M.D.

Iodide of potassium, the Iodkalium of the Germans, and the iodure de potassium of the French, is made by the addition of iodine in excess to an aqueous solution of potassa. It occurs in semi-opaque, white, transparent or colorless crystals, permanent in a dry air, rather deliquescent in a moist one, of an acrid saline taste. It is soluble in two-thirds its weight of water, and in six to eight parts of alcohol.

ANTAGONISTS AND INCOMPATIBLES.—It is incompatible, according to Biddle, with ammonium salts, sulphate, nitrate, phosphate, and borate of sodium, sulphates of potassium and magnesium, spirits of nitrous ether, soluble red salts and the mercurials generally; with chlorate of potassium, if a mineral acid is added, a poisonous iodate of potassium is formed. Therapeutically, its action is antagonized by all those remedies which promote constructive metamorphosis, and by the vaso-motor tonics, quinia, digitalis, cold, etc. (Wood). It should be remembered that calomel and potassium iodide exhibited together form the green iodide of mercury.

SYNERGISTS.—Alkalies and other remedies which promote waste favor the action of iodine and the iodides. Even mercury, which, theoretically, is incompatible with the iodide, is, especially in syphilis, synergistic.

PHYSIOLOGICAL ACTION.—When taken into the stomach the salt is rapidly absorbed and enters into the circulation. In the stomach it may cause, at first, a feeling of coolness, this being followed by warmth, or even burning, if the dose has been considerable. By many it is claimed that when the potassium salt enters the blood it is changed into a sodium salt. This theory, according to Ringer, has not yet been substantiated. Professor Binz ("Am. Jour. of the Med. Sci.," vol. lxx, p. 234) holds that, when the salt is taken into a healthy stomach, part is changed, by the action of the hydrochloric acid in that viscus, into hydriodic acid; another part is acted on by the chloride of sodium aud chauged into the iodide of sodium; and, if enough has been taken, the residue remains unchanged. All three combinations pass quickly into the circulation, when the hydriodic acid meets with soda and forms iodide of sodium; the iodide of sodium is acted on by carbonic and other acids, and iodine is set free. It has also been asserted that iodide of potassium is decomposed by the ozone, and depends for its action upon liberated iodine (Wood's "Therapeutics," 1876, p. 379). This theory also needs substantiation. By some of the leading authorities iodine and iodide of potassium are said to have the same action in the system, and many hold that the iodide depends for its action upon the iodine contained in it. It is true that both iodine and the iodide, when taken in sufficient doses, cause gastro-intestinal disturbance, and, when given continuously, produce iodism; but their theaapeutical uses are in many points different. Iodine acts upon the chronic enlargements of scrofulosis more effectively than the iodide, while the iodide is *par excellence* the rcmedy for the tertiary enlargements of syphilis. In rheumatic affections the iodide is employed, but here, no doubt, the iodide owes its efficacy, in good measure, to the potassium. The diuretic action of the iodide may also be ascribed to its potassium, though Bouchard affirms that iodine increases the elimination of urea.

The iodide of potassium exerts an alterative effect upon the tissues. In the physiological state the iodides increase waste and the elimination of the products of waste, and, if administered for a length of time, they cause emaciation and a general depression of the vital functions. Wood states that he has given the iodide in enormous doses, and seen nervous symptoms developed in only one case, where two hundred and seventy grains a day were exhibited, and the patient became intensely stupid and sleepy. An apparent contradiction to the depressing and emaciating effect of the drug is found in its action in syphilis, where, under its administration, the patients are seen to gain in health and strength. This action in syphilis is accounted for by the so-called specific action of the salt, the virus being eliminated by it, and the system at once reacting. Dr. C. Binz ("Grundzuge der Arzneimittellehre," Berlin, 1879) gives as a probable theory of the action of the iodide upon syphilitic and other abnormal deposits, the following: If iodide of potassium comes in contact, outside of the body, with protoplasm, water and carbonic acid, it is changed by the oxidizing prrperties of the protoplasm into bicarbonate of potassium and free iodine, this reaction being necessarily accompanied by some change in the protoplasm, yet undetermined. In the diseased body, if a pathological cell organization, water and carbonic acid, or some stronger acid, are together present (and, where abnormal cells are found, the other elements will not be difflcult to find); then the same reaction must take place in the presence of the potash salt, the elements of the protoplesm being broken up, the bicarbonate of potassium being formed and the iodine set free, which latter unites with the sodium of the blood and appears in the secretions as the iodide of sodium.

The salt is almost as rapidly eliminated as it is absorbed, so that, within fifteen minutes after taking it, it may be found in the saliva and the urine. The chief eliminator of the salt is the kidney, but the broncho-pulmonary, faucial and salivary glands take part in this action. Hence, we find, during a course of the iodide, that a stimulating effect is often exerted upon the mucous membrane of the lungs, fauces, kidneys, etc., a greater quantity of mucus being thrown from the lungs, symptoms of acute coryza not unfrequently showing themselves, and the taste of the iodide with an increased flow of saliva being noticed in the mouth.

IODISM.—A characteristic effect of the iodide, when given for a continued period, is the production of what has been called iodism: this it owes to its iodine. The quantity necessary to produce iodism varies with the susceptibility of the individual. Ringer states that in some peculiarly susceptible individuals one grain of the iodide, or even a part of a grain, has induced it. Most patients, however, bear the administration of the iodide without experiencing any unpleasant effects; and, usually, even if iodism is induced, the system soon accommodates itself to the drug, a state of tolerance being established, though no change may have been made in the dose. In some patients in whom it is desirable to induce iodism, enormous doses have to be administered before the required effect is produced.

The symptoms of iodism are general malaise, frontal headache, coryza, lachrymation, sometimes inflammatory swelling of the eyelids, bitter saline taste in the mouth, sore throat, hoarseness and dysphagia. An eruption of acne, especially on the face, shoulders and thighs, is a common result of the continuous use of the iodide, and it may be present indepently of any other symptom of iodism. Duhring says that "iodide of potassium may give rise to erythematous, papular, vesicular, pustular, bullous and purpuric lesions. The erythematous efflorescence, not very uncommon, occurs usually upon the forearms in discrete or confluent patches, and also upon the face and neck. If the administration of the drug is persisted in, this may go on to the papular form, which is rarer. The vesicular or eczema-form variety occurs in patients who have long continued the use of the drug; by some it is said to be most common on the scalp and scrotum; others describe its occurrence on the chest or limbs, and as accompanied by severe itching and desquamation." One case is reported (Bumstead and Taylor, "Venereal Diseases," New York, 1879) where only moderate doses caused an eruption like eczema rubrum over the whole body, accompanied by fever, some dyspnœa, and copious exudation. The pustular eruption bears a close resemblance to that caused by bromide of potassium, and is acneform in appearance. This is the acne eruption before referred to. Sometimes the pustules are followed by indurations which may persist. There is also a rare form of vesiculo-pustular eruption described by Duhring ("Diseases of the Skin," 1881, p. 329) which resembles an irritated patch of ring-worm. The bullous eruption occurs most frequently about the head, neck and upper extremities, less frequently upon the lower limbs, rarely upon the trunk. The eruption begins as pin-point-sized vesicles, or as shot-like papules, at the apices of which vesiculation appears, the vesicles being of a pale, yellowish-white color. If the iodide be given in large doses or continued, the blebs become dark red ar purplish, the serum, at first clear, becoming puriform and sanguinolent. Upon the discontinuance of the iodide, the lesions usually disappear within a few days or a week. Purpura is rarely produced by the iodide. It commonly appears soon after the beginning of a course of the medicine, and is most apt to occur upon the legs, less frequently upon the neck, face and other parts of the body; taking in some cases the miliary form, sometimes occurring in large patches, and it may even take on the form of purpura hemorrhagica. Hydroa, says Hutchinson, is most frequently caused by the iodide. It has, also, been asserted that atrophy of the testes and mammæ takes place under the use of the iodide, but many prominent writers, as Bartholow, Van Buren and Keyes, and others, deny this. That it has an antaphrodisiac effect, and, in long-continued doses, is capable of causing loss of sexual power, is, according to Bartholow, quite certain.

Some cases of iodism are characterized by nervous troubles—neuralgia, ringing in the ears, convulsions, disturbances of vision, and paralysis. Rilliet (Trousseau's report on memoir, "Bull. de l'Acad. Roy.," xxv) says the iodic cachexia is most easily induced in goitrous persons, and is characterized by rapid emaciation, commencing mostly in the face, severe nervous palpitations of the heart and excessive appetite. If the drug is continued. hysteria or hypochondriasis, with insomnia, manifests itself.

TREATMENT OF IODISM.—The unpleasant effects of the iodide appear earlier and continue longer in those in whom the processes of elimination are deficient or slow. If large draughts of water are taken with the iodide, in many cases iodism may be prevented, the water aiding in elimination. Bumstead states that if Fowler's solution is administered with the iodide, the eruption of acne may be prevented. Some claim that, if a full dose of carbonate or spirits of ammonia be administered with the iodide, the unpleasant effects of iodism may be obviated; but Ringer states that he has many times tried this, with no decided effects. If, on continuing the drug, the state of tolerance is not established, and if, after exhibiting it highly diluted, or with ammonia, the bad symptoms still continue, by stopping the drug for a few days they will all disappear without any other treatment.

ADMINISTRATION.—The dose of iodide of potassium is usually, to begin with, five to fifteen grains. Its limit is, practiclly, as much as will be tolerated by the system, or enough to produce the desired effect. It may be given pure or in solution, preferably the latter, and the higher the dilution the better. Seguin ("Arch. of Med.," vol. vi, 1881, p. 34) gives the following directions to prevent any gastric disturbance arising from the use of the salt: 1. Use a simple aqueous solution. 2. Give it upon an empty stomach. fifteen or thirty minutes before the ingestion of food. 2. Give it freely diluted with an alkaline solution. He uses a solution of equal parts of the iodide and water, by waight, and finds that a loss of one-fifth is made by mixing the salt and water, so that in one hundred drops of the solution there are only about eighty grains of the iodide. As an alkaline diluent, he advises Vichy water, one-half or one glassful being taken with each dose of the salt. If neither the Vichy nor the Vichy effervescent salt can be procured, then a pinch of bicarbonate of sodium to a glassful of water will answer.

THERAPEUTICS.—As the object of this paper is principally to discuss the use of iodide of potassium in syphilis, the writer will merely mention that the salt is useful in the following maladies, viz.: bronchocele, scrofula, chronic rheumatism, gout; as a diuretic and alterative in Bright's disease (fibroid degeneration), in spasmodic asthma, acute coryza, acute and chronic bronchitis, hay asthma, chronic pleurisy, chronic pericarditis, chronic splenitis, chronic laydrocephalus, basilar meningitis, aneurism; and as an eliminative in mercurial and saturnine poisoning. Locally, it forms an excellent application in aphthæ, mercurial stomatitis, pharyngitis and tonsillitis.

But it is the part the iodide plays in the treatment of syphilis which makes its study of special interist to the syphilographer. It has been used during the presence of the initial lesion, during the prodromal fever, and during the whole secondary or tertiary stage of the disease. M. F. Diday ("Therap. de Mal. Ven. et des Mal. Cutan.," Paris, 1876, p. 269) gives the salt during the presence of the chancre, before the stage of eruption (secondary), "not to drive out the poison of the virus, but to brace the constitution against it." To this end he gives, every morning and evening, one tablespoonful of a mixture containing: distilled water, gr. 500; iodide of potassium, gr. 12; citrate of iron, gr. 2, giving it freely diluted in water. He uses the same mixture during the prodromal fever He claims that the good results from this method of treatment are due to the action of the iodide upon the red blood globules, saying: "M. Grassi has determined by his experiments that, during the chancre, the proportion of the red blood globules is diminished by about one-quarter; that the iodide of potassium administered during this time re-established their normal proportion in fifteen or twenty days; and that, further, the iodide of mercury is far from producing the same good effects." Most authorities, however, state that the iodide is not of much use in the first stage of syphilis, nor in the early part of the second stage, but that it is most useful in the so-called tertiary lesions. Van Buren and Keyes give, as a rule for the time of its administration, the following: "As soon as the cutaneous lesions show a marked tendency to aggregate in patches, and especially to remain long chronic as scaly or tubercular thickened patches, or, indeed, without eruption after the first year of syphilis, the iodide should be given in the mixed treatment" (Van Buren and Keyes, "Genito-urinary Diseases, with Syphilis," New Nork, 1877, p. 565). The indication for giving the drug is rather the character of the syphilitic lesion, and the condition of the patient in regard to his having taken mercury, than the time which the disease has lasted. All authorities concur in stating that it gives its most brilliant results in the ulcerating and the gummy forms of syphilis, and in the deep lesions of bone, brain and viscera. It is especially useful when the syphilitic or mercurial cachexia is present, or when the patient has taken mercury for some time without effect. Niemeyer says that "the iodide is indicated and gives most relief in all cases where the spontaneous extinction of the malady is not to be calculated on, and in which the employment of mercury is contraindicated." Bartholow, ("Materia Medica and Therapeutics," New York, 1877, p. 182) says: The iodide of potassium is useful and curative in syphiloma of the nervous system, in mental disorders, epileptiform seizures, paralytic states, etc., dependent on gummata, nodes, etc., in neuralgia of the fifth, nocturnal pain in the head, syphilitic paraplegia, the various neuralgiæ of syphilis, ulceration of the nares, palate, tonsils and larynx, syphilitic deposits in the lungs simulating phthisis, syphilitic disease of the spleen, liver, kidneys and other viscera, syphilitic rheumatism and the late skin eruptions of syphilis. Rosenthal ("Nervenkrankheiten," Wien, 1875, p. 234) prefers mercury to the iodide in syphilitic disease of the brain.

The iodide, when administered for these late syphilitic lesions, should be pushed rapidly till the full effects of the drug are manifested, or till iodism is induced. Unfortunately, while the salt undoubtedly relieves the urgent symptoms, it does not possess those truly curative or specific qualities of mercury; so that, after relief is obtained by its use, mercury must be again resorted to for a permanent cure, the two drugs being continued in combination.

When the iodide is given after a course of mercury, sometimes salivation is induced, it having the property of rendering mercury, or any of its compounds retained within the tissues of the body, soluble, and of throwing them back into the circulation, to be eliminated by the kidneys. It has been affirmed that any good which may result from the administration of the iodide is due to its solvent power upon the mercury held in combination with the albuminoids of the body.

It is quite customary and really useful to administer the iodide with mercury in the so-called "mixed treatment," notably in the later stages of syphilis. There have been combinations of nearly all the preparations of mercury with the iodide, but probably the most used are the bichloride and the biniodide. The solution of the bichloride with the iodide is chemically unsound, the biniodide of mercury being formed with iodide ef potassium in excess. It is, nevertheless, useful. As a vehicle, perhaps, the compound syrup of sarsaparilla is as good as any, the English authors speaking highly of it for its own sake, and it is certainly a pleasant medium. Lee ("Lectures on Syphilis," London, 1875) thinks that if the iodide of potassium is given by the mouth, and mercury by inunction, they unite in the system and produce about the same effect as if iodide of mercury were given internally. This is a good way to treat chronic cases where the stomach must be spared as much as possible.—*N. Y. Med. Jour., Oct. 1882.*

